# Empagliflozin maintains capillarization and improves cardiac function in a murine model of left ventricular pressure overload

**DOI:** 10.1038/s41598-021-97787-2

**Published:** 2021-09-15

**Authors:** Masaaki Nakao, Ippei Shimizu, Goro Katsuumi, Yohko Yoshida, Masayoshi Suda, Yuka Hayashi, Ryutaro Ikegami, Yung Ting Hsiao, Shujiro Okuda, Tomoyoshi Soga, Tohru Minamino

**Affiliations:** 1 Department of Cardiovascular Biology and Medicine, Niigata University Graduate School of Medical and Dental Sciences, Niigata, 951-8510 Japan; 2 Department of Cardiovascular Biology and Medicine, Juntendo University Graduate School of Medicine, 2-1-1 Hongo, Bunkyo-ku, Tokyo, 113-8421 Japan; 3 Division of Bioinformatics, Niigata University Graduate School of Medical and Dental Sciences, Niigata, 951-8510 Japan; 4 Institute for Advanced Biosciences, Keio University, Yamagata, 997-0052 Japan; 5 Agency for Medical Research and Development-Core Research for Evolutionary Medical Science and Technology (AMED-CREST), Agency for Medical Research and Development, Tokyo, 100-0004 Japan

**Keywords:** Heart failure, Cardiac hypertrophy

## Abstract

Patients with type 2 diabetes treated with Sodium glucose transporter 2 (SGLT2) inhibitors show reduced mortality and hospitalization for heart failure (HF). SGLT2 inhibitors are considered to activate multiple cardioprotective pathways; however, underlying mechanisms are not fully described. This study aimed to elucidate the underlying mechanisms of the beneficial effects of SGLT2 inhibitors on the failing heart. We generated a left ventricular (LV) pressure overload model in C57BL/6NCrSlc mice by transverse aortic constriction (TAC) and examined the effects of empagliflozin (EMPA) in this model. We conducted metabolome and transcriptome analyses and histological and physiological examinations. EMPA administration ameliorated pressure overload-induced systolic dysfunction. Metabolomic studies showed that EMPA increased citrulline levels in cardiac tissue and reduced levels of arginine, indicating enhanced metabolism from arginine to citrulline and nitric oxide (NO). Transcriptome suggested possible involvement of the insulin/AKT pathway that could activate NO production through phosphorylation of endothelial NO synthase (eNOS). Histological examination of the mice showed capillary rarefaction and endothelial apoptosis after TAC, both of which were significantly improved by EMPA treatment. This improvement was associated with enhanced expression phospho-eNOS and NO production in cardiac endothelial cells. NOS inhibition attenuated these cardioprotective effects of EMPA. The in vitro studies showed that catecholamine-induced endothelial apoptosis was inhibited by NO, arginine, or AKT activator. EMPA activates the AKT/eNOS/NO pathway, which helps to suppress endothelial apoptosis, maintain capillarization and improve systolic dysfunction during LV pressure overload.

## Introduction

The outcome of the Empagliflozin Cardiovascular Outcome Event Trial in Type 2 Diabetes Mellitus Patients (EMPA-REG) clearly showed that SGLT2 inhibitors suppress pathologies in heart failure^1^. In the EMPA-REG, diabetic patients receiving empagliflozin (EMPA) showed lower risk for composite cardiovascular outcome and death from any cause than patients receiving standard diabetes care^1^. These findings were confirmed by the Canagliflozin Cardiovascular Assessment Study (CANVAS)^2^ and Dapagliflozin Effect on Cardiovascular Events-Thrombolysis in Myocardial Infarction 58 (DECLARE-TIMI 58) trial^3^, both of which studied different types of SGLT2 inhibitors. More recently, the Dapagliflozin and Prevention of Adverse Outcomes in Heart Failure (DAPA-HF) trial and the Empagliflozin Outcome Trial in Patients with Chronic Heart Failure and a Reduced Ejection Fraction (EMPEROR-Reduced) showed that treatment with SGLT2 inhibitors significantly reduced the risk of worsening heart failure or death from cardiovascular causes, regardless of the presence or absence of type 2 diabetes^4,5^. Nowadays, the cardioprotective effects of SGLT2 inhibitors are well recognized as a “class effect.” The biological effects of these diabetic drugs are considered to be mediated by several mechanisms, including hemodynamic effects, ketone production, inhibition of sodium-hydrogen exchange in myocardial cells, and reduced adrenergic tone; however, the underlying mechanisms are not fully understood^6,7^. Vascular endothelial growth factor-A (VEGF-A), one of the main angiogenic factors, was previously reported to be reduced in a murine left ventricular (LV) pressure overload model^8^. The lower levels of VEGF-A led to capillary rarefaction and tissue hypoxia in the failing heart and caused the progression of systolic dysfunction^8,9^. Here, we show that in hearts subjected to LV pressure overload EMPA maintains capillarization by activating the AKT/eNOS/NO pathway in endothelial cells (ECs), which helps to suppress endothelial apoptosis and enhance cardiac systolic function.

## Results

### EMPA ameliorates systolic dysfunction in murine LV pressure overload model

**Figure 1:**
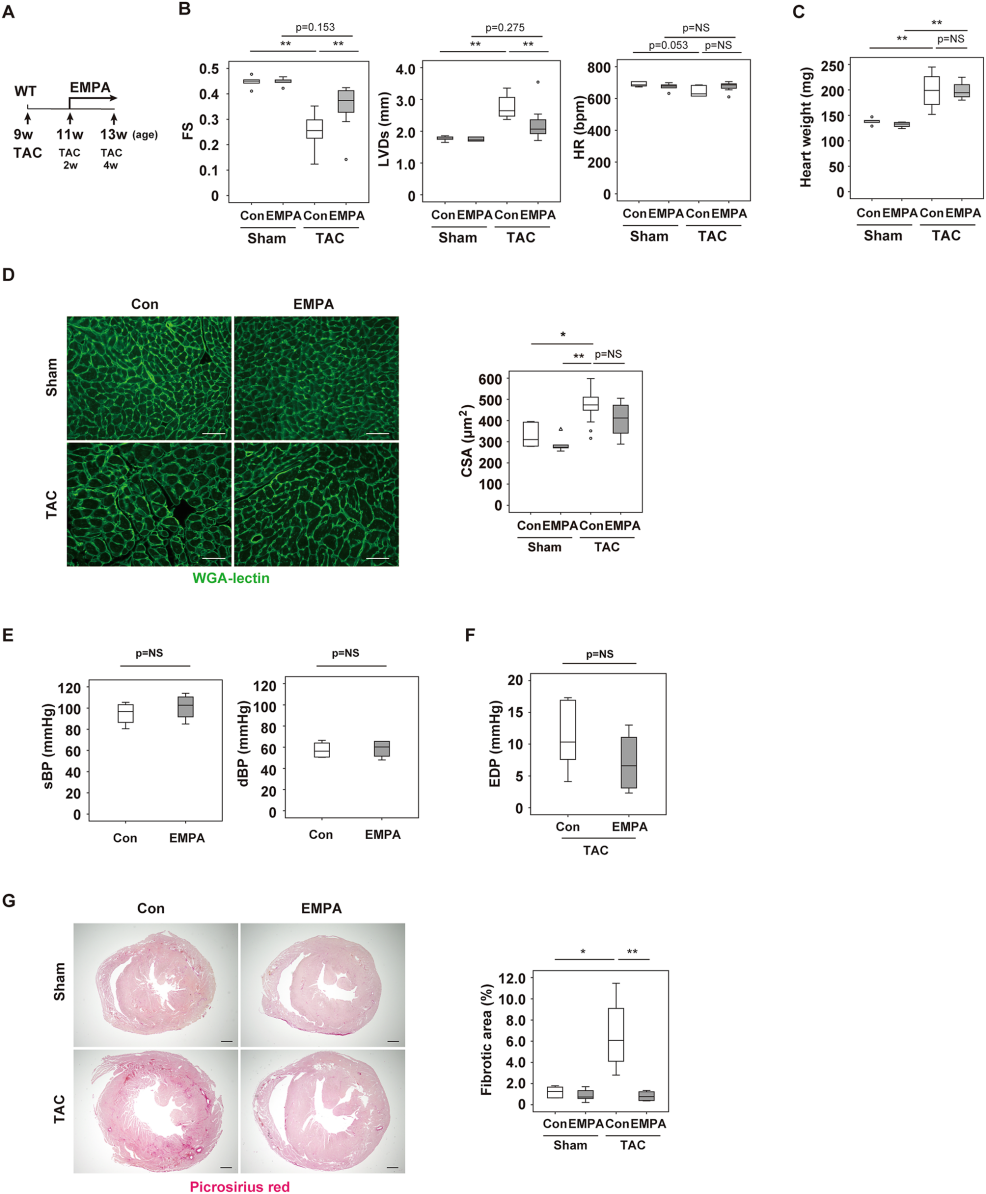
Characterization of heart with empagliflozin administration. (**A**) Protocol of empagliflozin(EMPA) administration. At 9 weeks of age, mice were subjected to TAC operation. Two weeks after TAC, some mice were subjected to EMPA administration for totally 2 weeks and euthanized at 13 weeks of age (4 weeks after TAC operation). (**B**) Echocardiographic data analyzing fractional shortening (FS), left ventricular systolic dimension (LVDs) or heart rate (HR) of mice subjected to TAC or Sham operation with or without (Con) administration of empagliflozin (EMPA) (n = 5, 5, 8, 7). For this study, 1 outlier (in the TAC + EMPA group) was excluded by boxplot (SPSS) for further statistical analysis (n = 5, 5, 8, 6 were analyzed) (only for FS and LVDs studies). (**C**) Heart weight of indicated mice (n = 5, 5, 4, 4). For this study, 2 outliers (in the Sham + Con group) were excluded by boxplot (SPSS) for further statistical analysis (n = 3, 5, 4, 4 were analyzed). (**D**) Wheat germ agglutinin (WGA) staining of left ventricle (LV) in indicated mice. Scale bar = 50 μm. Right panel indicates cross sectional area (CSA) of cardiomyocytes of indicated group (n = 6, 5, 13, 5). (**E**) Systolic (sBP; n = 4, 4) or diastolic (dBP; n = 4, 4) blood pressure of indicated mice. (**F**) Left ventricle end diastolic pressure (EDP) of indicated mice (n = 6, 6). (**G**) Picrosirius red staining of LV in indicated mice. Scale bar = 500 μm. Data were analyzed by a 2-way analysis of variance (ANOVA) followed by Tukey’s multiple comparison test (**B**–**D**), by a 2-tailed Student’s *t* test (**E**,**F**), or by non-parametric Kruskal Wallis test (**G**). *P < 0.05, **P < 0.01. Values are shown as the mean ± SEM. NS = not significant. Small circle indicates outlier; triangle indicates abnormal value. Analyses were performed with and without these values; when the results of both analyses were nonsignificant, the difference was described as NS.

In the LV pressure overload model, systolic function was reduced and cardiac dilatation was present 4 weeks after TAC. Administration of EMPA (TAC + EMPA) ameliorated these phenotypes (Fig. 1A,B). Body and heart weight were similar between the TAC + Control and TAC + EMPA groups (Fig. 1C and Supplemental Fig. S1A). Staining with HE showed no marked difference in the cardiac sections between these groups (Supplemental Fig. S1B), and the cross-sectional area of the cardiomyocytes was similar between the TAC + Control and TAC + EMPA groups (Fig. 1D). Both systolic and diastolic blood pressure were comparable between the Control and EMPA groups (Fig. 1E). End diastolic pressure of the left ventricle tended to be lower in the TAC + EMPA group than in the TAC + Control group, but this difference was not statistically significant (Fig. 1F). In both the Sham + EMPA and the TAC + EMPA groups, the volume of urine was higher than in the respective control group and the concentration of renal sodium was lower (Supplemental Fig. S1C). Fractional excretion of sodium (FENa), plasma creatinine, and creatinine clearance (CCr) were similar among the groups (Supplemental Fig. S1C,D). The fibrotic area was significantly lower in the TAC + EMPA group than in the TAC + Control group (Fig. 1G).

### EMPA increases citrulline levels in cardiac tissues and plasma, but this increase does not mediate cardioprotective effects

**Figure 2:**
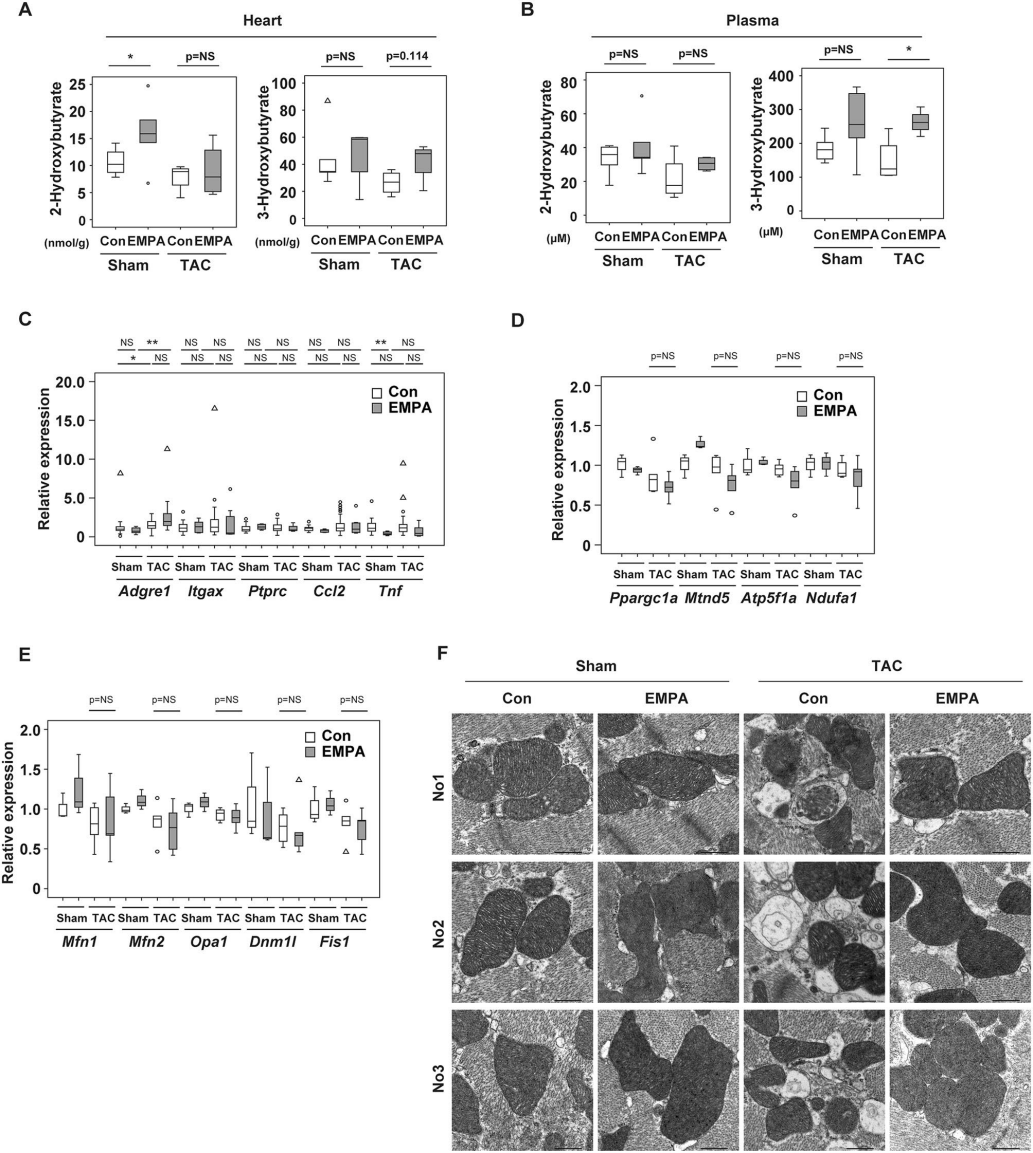
Level of plasma 3-Hydroxybutyrate level with EMPA. (**A**,**B**) Level of 2-hydroxybutyrate or 3-hydroxybutyrate (n = 5, 5, 4, 4) in heart (**A**) or plasma (**B**) of indicated mice. (**C**–**E**) Quantitative polymerase chain reaction (PCR) of *Adgre1* (n = 28, 8, 31, 8)*, Itgax* (CD11c) (n = 25, 8, 30, 7), *Ptprc* (CD45) (n = 24, 8, 28, 7)*, Ccl2* (n = 28, 8, 31, 9)*,* and *Tnf* (n = 27, 8, 29, 8; **C**); *Ppargc1a, Mtnd5, Atp5f1a,* and *Ndufa1* (n = 3, 3, 5, 5; **D**); and *Mfn1, Mfn2, Opa1, Dnm1l,* and *Fis1* (n = 3, 3, 5, 5; **E**) of indicated mice. (**F**) Electron microscopic findings of left ventricle (LV) of indicated mice. No1, No2, and No3 indicate that samples originate from different biological replicates. Scale bar = 2 μm. Data were analyzed by a 2-tailed Student’s *t* test (**A**,**B**), or by a 2-way analysis of variance (ANOVA) followed by Tukey’s multiple comparison test (C(*Itgax*, *Ptprc*), **D**, **E**), by Kruskal Wallis (C(*Adgre1*, *Tnf*)), or by Dunnett correction (C(*Ccl2*)). The following were excluded for further analyses: in (**A**), 2 outliers in Sham + EMPA group. *P < 0.05, **P < 0.01. Values are shown as the mean ± SEM. *NS* not significant. Small circle indicates outlier; triangle indicates abnormal value. Analyses were performed with and without these values; when the results of both analyses were nonsignificant, the difference was described as NS.

SGLT2 inhibitors are thought to mediate their beneficial biological effects partly through favorable metabolic remodeling. We therefore conducted metabolomic studies in plasma and cardiac tissues of our heart failure model and found that hydroxybutyrates were increased in heart and plasma in the EMPA group (Fig. 2A,B), which is consistent with the previous studies^10–12^. Quantitative polymerase chain reaction (PCR) studies showed similar expression of inflammatory molecules, including *Adgre1* (Emr1), *Itgax* (CD11c), *Ptprc* (CD45), *Ccl2*, and *Tnf*, between the groups (Fig. 2C). Transcripts for mitochondria-related molecules, including *Ppargc1a*, *Mtnd5*, *Atp5f1a*, and *Ndufa1* (Fig. 2D), and mitochondrial dynamics, such as *Mfn1*, *Mfn2*, *Opa1*, *Dnm1l*, and *Fis1*, were similar between the groups (Fig. 2E). Electron microscopic findings showed that TAC led to an increase in autolysosomes in the left ventricle and that this increase was attenuated by EMPA administration (Fig. 2F). Hydroxyproline was reduced in both heart and plasma of the TAC + EMPA group compared with the TAC group (Supplemental Fig. S2A). We also found that some other metabolites were reduced or increased in the sham- or TAC-operated mice administered EMPA (Supplemental Fig. S2B,C). Citrulline was increased in both heart and plasma of mice administered EMPA (Supplemental Fig. S3A-C). Therefore, we decided to study this metabolite further because it can fuel the TCA cycle via the production of fumarate^10^ and activate metabolism in cardiomyocytes. In line with this concept, we found that administration of citrulline increased oxygen consumption in differentiated myocytes (Supplemental Fig. S3D), which led us to test the effect of this metabolite in our TAC model. Administration of citrulline did not ameliorate cardiac systolic dysfunction, and LV dilatation was not improved under this condition (Supplemental Fig. S3E-G). Heart weight was similar between the groups (Supplemental Fig. S3H), and HE and picrosirius staining to analyze the level of fibrosis showed no marked change with citrulline treatment (Supplemental Fig. S3I,J). Quantitative PCR studies showed that expression of some inflammatory molecules, including *Adgre1* and *Ptprc*, was decreased by citrulline treatment, whereas expression of *Ccl2* and *Tnf* did not differ between the groups (Supplemental Fig. S3K). Expression of mitochondria-related molecules, such as *Ppargc1a*, *Mtnd5*, *Atp5f1a*, and *Ndufa*, and mitochondrial dynamics, including *Mfn1*, *Mfn2*, *Opa1*, *Dnm1l*, and *Fis1*, was also similar between the groups (Supplemental Fig. S3L,M). These results suggested that citrulline per se plays a minor role in EMPA-induced improvement of cardiac function.

### EMPA leads to enrichment in KEGG term related to insulin signaling pathway

**Figure 3:**
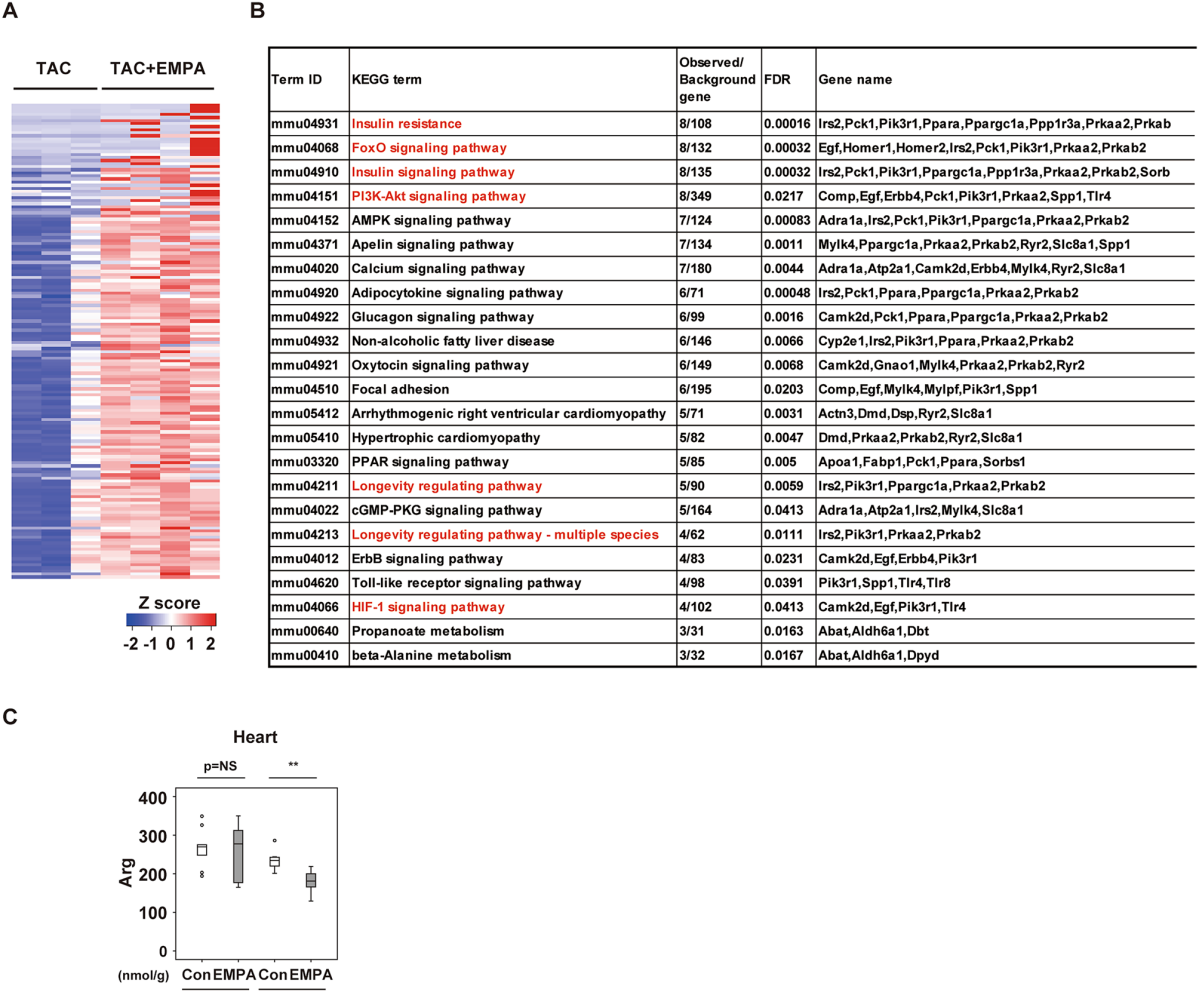
Results of RNA seq studies analyzing LV with EMPA administration. (**A**,**B**) RNA seq analysis data for cardiac tissues: Heat map (**A**) or KEGG categories enriched by twofold (q < 0.05, Benjamini–Hochberg correction for multiple-testing) in mice with empagliflozin (EMPA) administration and TAC (TAC + EMPA) as compared with mice with TAC only (**B**; n = 3, 4). For this study, cardiac tissues were collected 5 days after the introduction of EMPA at TAC2w, and samples were collected at TAC2w + 5 days. (**C**) Metabolomic study analyzing arginine (Arg) in cardiac tissues of indicated mice (n = 10, 10, 8, 9). For study in (**C**), 1 outlier (in TAC + Con group) was excluded by boxplot (SPSS) for further statistical analysis (n = 10, 10, 7, 9 were analyzed). Data were analyzed by 2-way analysis of variance (ANOVA) followed by Dunnett’s test (**C**). *P < 0.05, **P < 0.01. Values are shown as the mean ± SEM. NS = not significant. Small circle indicates outlier, triangle indicates abnormal value. Analyses were performed with and without these values; when the results of both analyses were nonsignificant, the difference was described as NS.

In the RNA-sequencing analysis in cardiac tissues, compared with the TAC group the EMPA + TAC group showed enrichment in KEGG terms^13^ related to insulin signaling, such as “insulin resistance,” “FoxO signaling pathway,” “Insulin signaling pathway,” “PI3K-Akt signaling pathway,” “Longevity-regulating pathway,” and “Longevity-regulating pathway–multiple species” (Fig. 3A,B). Quantitative PCR studies showed increases in the transcripts *Irs2*, *Pik3r1*, *Prkaa2*, and *Sorbs1* (Supplemental Fig. S4). In addition to the increased citrulline levels described above, we became interested in findings from metabolomic studies showing reduced arginine levels in cardiac tissues of the TAC + EMPA group compared with the TAC + Control group (Fig. 3C). Arginine is well known to be oxidized into citrulline and NO, and eNOS is known to mediate this reaction. AKT is one of the major signaling molecules in the insulin signaling pathway and is known to upregulate eNOS activity. Activation of eNOS was reported to mediate anti-apoptotic effects, and this activation ameliorated cardiac dysfunction in an ischemic heart failure model^14^. Capillary rarefaction was previously shown to develop with LV pressure overload and to contribute to the progression of reduced systolic function^8,9,15^. NO has a critical role in maintaining capillarization in tissues^16^, and because an SGLT2 inhibitor was reported to increase the phospho-AKT level in cardiac tissues of an ischemia–reperfusion injury model^17^ and activate AKT/eNOS signaling in HUVECs^18^, we speculated that EMPA treatment might activate AKT/eNOS signaling in the heart and attenuate capillary rarefaction in cardiac tissues, thereby improving systolic dysfunction after TAC.

### EMPA maintains capillarization in cardiac tissue during LV pressure overload

**Figure 4:**
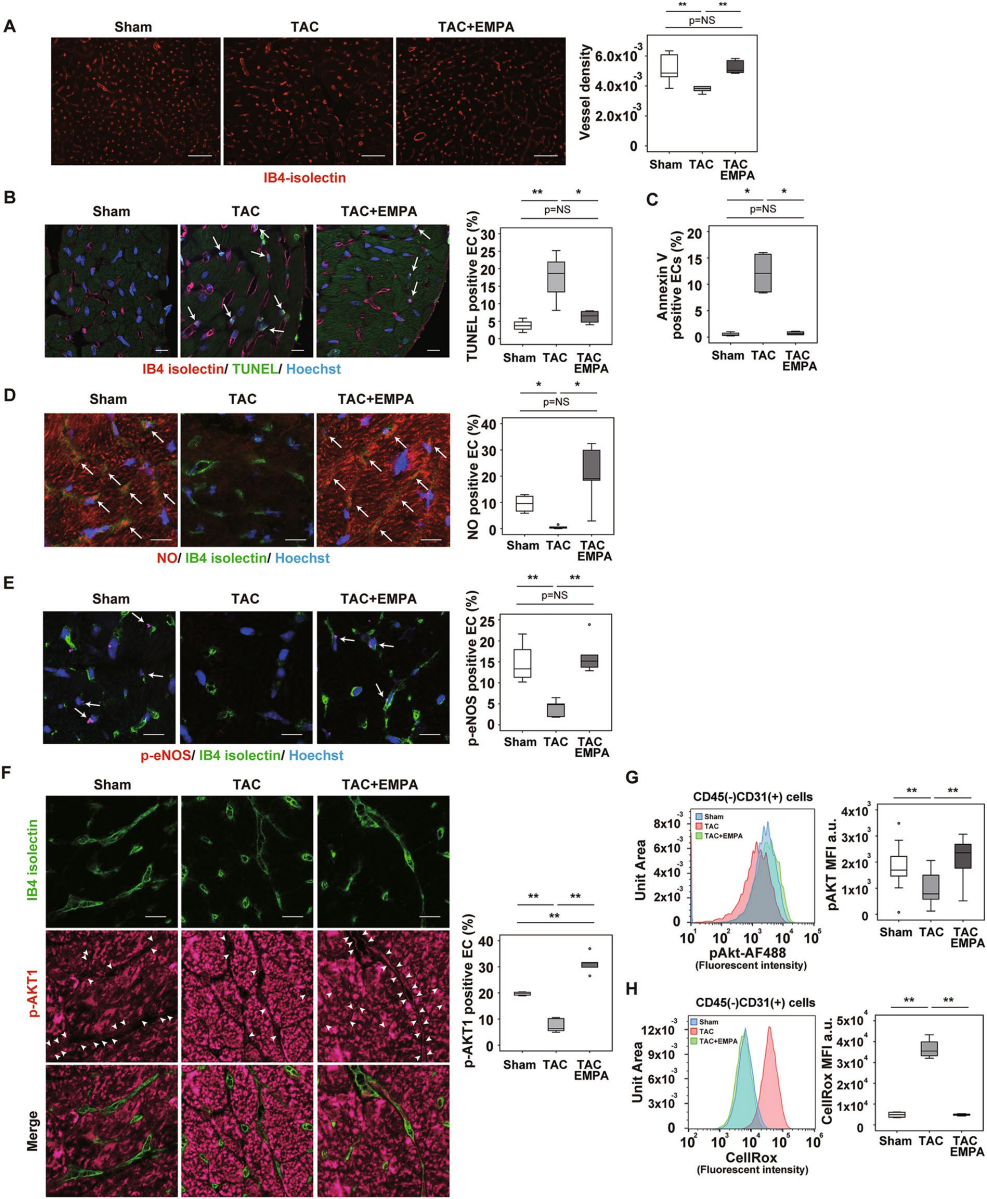
EMPA enhances capillarization in LV during pressure overload. (**A**) Capillarization of left ventricle (LV) of indicated mice as analyzed with IB4-isolectin staining. Right panel indicates vessel density (n = 6, 5, 5). Scale bar = 50 μm. (**B**,**D**–**F**): IB4-isolectin staining co-stained with TUNEL and Hoechst (**B**; scale bar = 10 μm; arrow indicates TUNEL-positive endothelial cells [ECs]); NO and Hoechst (**D**; scale bar = 10 μm; arrow indicates NO-positive ECs); p-eNOS (**E**; scale bar = 10 μm; arrow indicates p-eNOS positive ECs); and p-AKT1 (**F**; scale bar = 10 μm; arrow head indicates p-AKT1–positive ECs). Right panels indicate quantification (**B**, n = 4, 4, 4; **D**, n = 4, 5, 5; **E**, n = 4, 5, 5; and **F**, n = 4, 5, 5). (**C**,**G**,**H**) Flow cytometry studies analyzing level of Annexin V (**C**) (n = 4, 4, 4), pAKT (**G**) (n = 14, 14, 14), and reactive oxygen species (CellRox) (**H**) (n = 4, 4, 4) in CD45^-^CD31^+^ ECs extracted from cardiac tissues of indicated mice. Data were analyzed by 2-way analysis of variance (ANOVA) followed by Tukey’s multiple comparison test (**A**,**B**,**D**–**H**), or Kruskal Wallis correction (**C**). *P < 0.05, **P < 0.01. Values are shown as the mean ± SEM. *NS* not significant. Small circle indicates outlier, triangle indicates abnormal value. Analyses were performed with and without these values; when the results of both analyses were nonsignificant, the difference was described as NS.

Capillary network formation is critical for maintaining organ homeostasis. Suppression of VEGF-A was reported to induce capillary rarefaction in cardiac tissues of mice subjected to LV pressure overload and to promote cardiac remodeling. We confirmed that capillary rarefaction and reduced cardiac perfusion developed with LV pressure overload and that EMPA administration ameliorated these changes in cardiac tissues (Fig. 4A, and Supplemental Fig. S5A,B). Under this condition, levels of transcript for angiogenic form VEGF-A (*Vegfa164a*)^19^ in whole heart (Supplemental Fig. S5C) and in isolated endothelial cells (ECs) from cardiac tissues, defined as CD45^–^CD31^+^ cells, were similar in the TAC and TAC + EMPA groups (Supplemental Fig. S5D).

**Figure 5:**
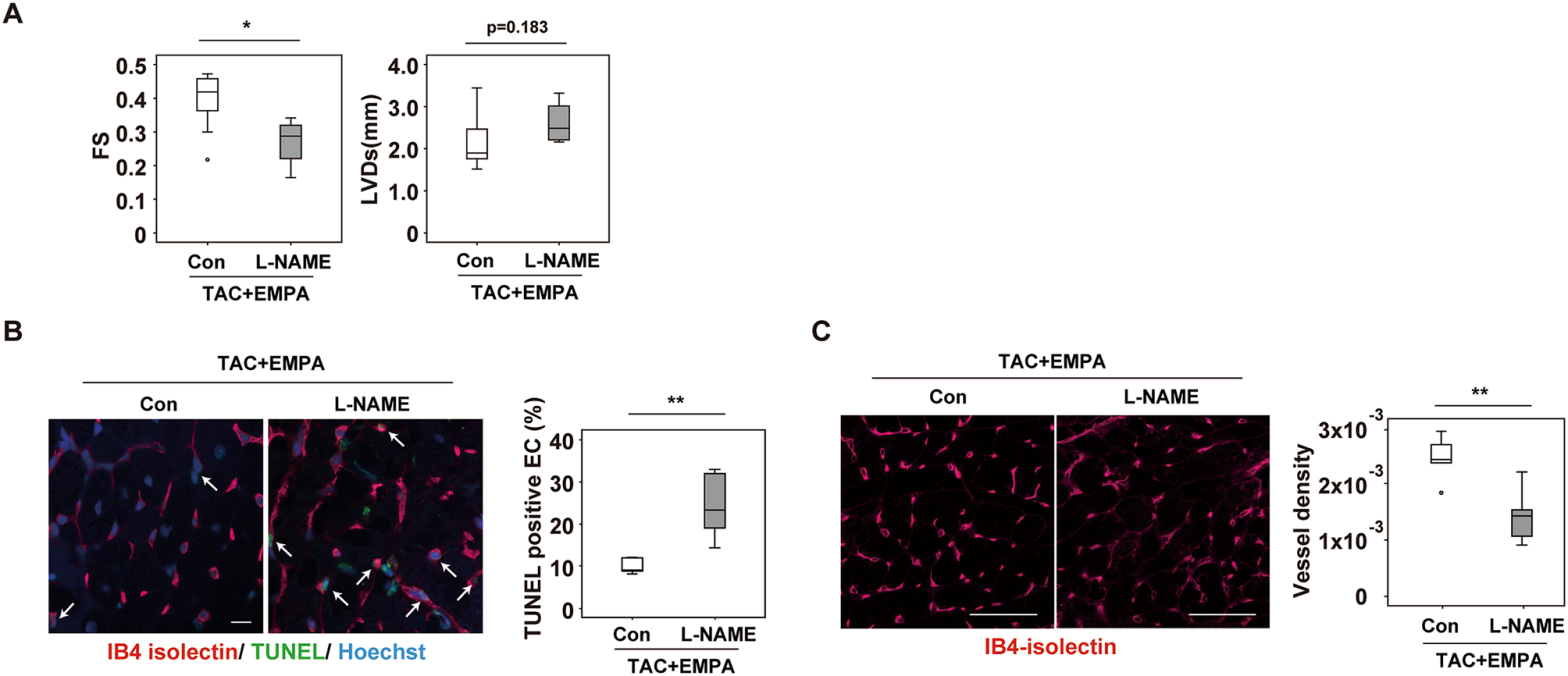
Effects of L-NAME administration. (**A**) Echocardiographic data analyzing fractional shortening (FS) or left ventricular systolic dimension (LVDs) of mice subjected to TAC + EMPA with or without L-NAME administration (n = 10, 4). (**B**) TUNEL (terminal deoxynucleotidal transferase–mediated biotin–deoxyuridine triphosphate nick-end labeling) of cardiac tissues of mice subjected to TAC + EMPA with or without L-NAME administration (arrow indicates TUNEL-positive ECs, scale bar = 10 μm) (right panel indicates quantification of TUNEL positive ECs (n = 6, 10)) (**C**) Immunofluorescence study for IB4-isolectin in cardiac tissues of indicated mice. Right panels indicates quantified data demonstrated as vessel density (estimated as the number of microvessels relative to the number of cardiomyocytes/cross sectional area (CSA) (μm^2^)) (n = 5, 10. scale bar = 100 μm). Data were analyzed by a 2-tailed Student’s *t* test (**A**–**C**). *P < 0.05, **P < 0.01. Values are shown as the mean ± SEM.

Adrenergic tone increases in patients with heart failure, and the catecholamine level predicts poor prognosis in these patients^20^. Catecholamine was previously reported to induce cell death in ECs^21^. We found that apoptotic ECs were increased in cardiac tissues after TAC, and this increase was attenuated by EMPA treatment (Fig. 4B,C, Supplemental Fig. S6A,B). Our metabolomic studies showed increased citrulline and reduced arginine levels in cardiac tissues of the TAC + EMPA group compared with the TAC group (Fig. 3C, Supplemental Fig. S3A,C), suggesting an enhancement of NO production by EMPA administration. Because NO was reported to suppress apoptosis^22^, we examined a possible role of the NO pathway in EMPA-induced suppression of EC apoptosis. The level of endothelial NO in cardiac tissues was reduced in the TAC group, and this reduction was significantly improved by EMPA administration (Fig. 4D and Supplemental Fig. S6C). Phosphorylated eNOS represents the activated form of this enzyme and has a critical role in the production of NO. We found that phosphorylated eNOS in ECs was reduced in the TAC group, and that this reduction was significantly improved by EMPA administration (Fig. 4E and Supplemental Fig. S6D), indicating that an EMPA-induced increase of phosphorylated eNOS upregulates NO production. To elucidate the upstream signal involved in the activation of eNOS by EMPA, we focused on AKT. AKT has a critical role for the phosphorylation and activation of eNOS, and SGLT2 inhibitors including EMPA and phlorizin were shown to increase phospho-AKT (S473), an activated form of AKT^18,23^. We found that the level of phospho-AKT was significantly higher in the TAC + EMPA group than in the TAC group (Fig. 4F,G). In the TAC group, endothelial cells showed a significant increase in reactive oxygen species (ROS), and this increase was attenuated by EMPA treatment (Fig. 4H). ROS was previously reported to inactivate AKT^24^, and our findings indicated a role of EMPA in the suppression of ROS-induced inactivation of AKT in these cells. Co-administration of an NOS inhibitor, L-NAME, promoted endothelial cell death, reduced capillarization, and suppressed cardiac function in EMPA-treated TAC mice (Fig. 5A–C), suggesting a causative role of the eNOS/NO pathway in mediating the cardioprotective effects of EMPA.

**Figure 6:**
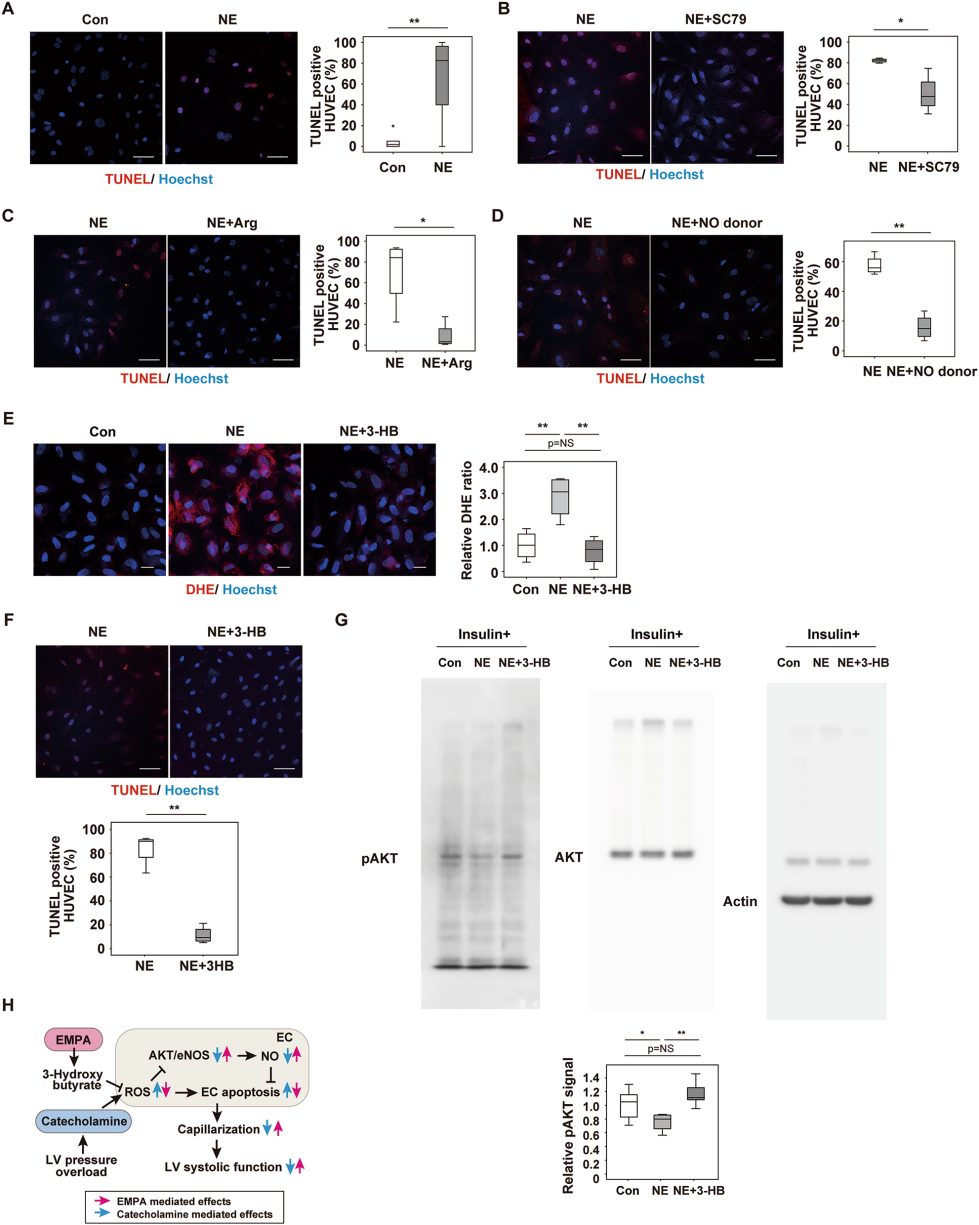
The 3-Hydroxybutyrate suppresses ROS and apoptosis in endothelial cells. (**A**–**D**,**F**):, human umbilical vein endothelial cells (HUVECs) with addition of phosphate-buffered saline (Con) or norepinephrine (NE) (**A**; n = 6, 14; scale bar = 50 μm); NE or NE + SC79(AKT activator) (**B**; n = 4, 4; scale bar = 50 μm); NE or NE + Arg (**C**; n = 4, 4; scale bar = 50 μm); NE or NE + NO donor (**D**; n = 4, 4; scale bar = 50 μm), or NE or NE + 3-Hydroxybutyrate (3-HB) (**F**; n = 4,4; scale bar = 50 μm). (**E**) Dihydroethidium (DHE) staining of HUVECs administrated with NE or NE + 3-Hydroxybutyrate (3-HB) (scale bar = 10 μm), and the quantified data analyzed as relative DHE ratio (DHE area was divided with nuclear number/view, and subjected to further quantification studies) (n = 4, 4, 4). (**G**) Western blot detecting signals from pAKT, AKT or beta Actin (Actin) in HUVECs administrated with insulin together with NE or NE + 3-HB. Right panel is the quantified data showing relative pAKT signal (n = 6, 6, 6) (signal was analyzed as pAKT/AKT/Actin). (**H**) Scheme summarizing our findings. Empagliflozin (EMPA) activates the AKT/eNOS/NO pathway and suppresses EC apoptosis through an increase of 3-Hydroxybutyrate and reduction of reactive oxygen species (ROS). This change leads to enhancement of capillarization in the left ventricle (LV) during pressure overload and ameliorates cardiac dysfunction. Data were analyzed by a 2-tailed Student’s *t* test (**A**–**D**,**F**), or by 2-way analysis of variance (ANOVA) followed by Tukey’s multiple comparison test (**E**,**G**). *P < 0.05, **P < 0.01. Values are shown as the mean ± SEM. *NS* not significant. Small circle indicates outlier, triangle indicates abnormal value. Analyses were performed with and without these values; when the results of both analyses were nonsignificant, the difference was described as NS.

In vitro studies showed that norepinephrine (NE) administration promoted apoptosis in HUVECs (Fig. 6A), and NE-induced apoptosis was suppressed by co-administration of an AKT activator (SC79) (Fig. 6B), arginine (Fig. 6C), or an NO donor (Fig. 6D). It is well accepted that butyrate mediates the beneficial effects of SGLT2 inhibitors^25,26^, and in our study 3-HB showed a marked increase in the plasma of the TAC + EMPA group (Fig. 2B). In HUVECs, NE increased the ROS level, and this increase was suppressed by 3-HB treatment (Fig. 6E); 3-HB treatment also reduced NE-induced apoptosis in HUVECs (Fig. 6F). In HUVECs, NE administration reduced the pAKT level, and this reduction was attenuated by 3-HB administration (Fig. 6G). These results suggest that EMPA inhibits catecholamine-induced downregulation of the AKT/eNOS/NO pathway by increasing hydroxybutyrate, which could suppress catecholamine-induced increase of ROS. Upregulation of the AKT/eNOS/NO pathway would suppress endothelial apoptosis, maintain capillarization and improve systolic dysfunction during LV pressure overload.

We conducted temporal studies (Supplemental Fig. S7A) and found that EMPA administration inhibited the progression of systolic dysfunction in TAC mice (Supplemental Fig. S7B). In contrast, systolic function appeared to decrease progressively in the TAC + Control group, although the change from TAC 2w to TAC 4w was not statistically significant. Likewise, LV dilatation significantly increased from TAC 2w to TAC 4w in the TAC + Control group but not in the TAC + EMPA group. Better cardiac function was observed as early as 1 week after EMPA treatment, and this improvement was associated with increased capillary density and reduced endothelial apoptosis (Supplemental Fig. S7C,D). These results suggest that EMPA treatment could stop the progression of cardiac pathology during pressure overload rather than restore cardiac function.

Taken together, our results indicate that EMPA administration activates the AKT/eNOS/NO pathway and contributes to the suppression of catecholamine-induced apoptosis in ECs and the enhancement of capillarization in heart, leading to improvement of cardiac dysfunction in our TAC model (Fig. 6H). These findings showed the previously unknown cardio-protective role of EMPA during LV pressure overload.

## Discussion

The outcome of EMPA-REG opened a new avenue for cardiovascular-metabolic research^1^. Another line of evidence also indicated that SGLT2 inhibitors ameliorate pathologies related to heart failure in patients with type 2 diabetes^2,3^. In these studies, patients treated with SGLT2 inhibitors had a lower risk for hospitalization due to heart failure and a lower risk of death. SGLT2 inhibitors were tested in patients with nondiabetic heart failure and resulted in similar beneficial cardiovascular outcomes^4^. This finding led to the repurposing of this diabetes drug to combat heart failure, and recently the Food and Drug Administration (FDA) in the USA approved an SGLT2 inhibitor for the treatment of heart failure with reduced ejection fraction (HFrEF) in adults with or without diabetes^5^.

A fundamental question in this context is the possible mechanisms underlying the effects of SGLT2 inhibitors. Evidence indicates that the beneficial biological effects of these drugs may be mediated by several mechanisms. For example, hemodynamic effects and increased ketone production, inhibition of sodium-hydrogen exchange in myocardial cells, enhancement of myocardial oxidative phosphorylation, and reduction of adrenergic tone were reported to be cardioprotective^6,12,27–29^. The lack of knowledge about the underlying mechanisms motivated us to look for other potential pathways that may be involved; however, the search for new molecular mechanism proved to be challenging. As shown in Figs. 1 and 2, our initial studies, which included electron microscope investigations, were not informative or helpful. As shown in Supplemental Fig. S3, we then performed metabolomic studies and found that EMPA administration increased citrulline in both heart and plasma. Citrulline has the potential to fuel TCA, and in vitro studies that examined myocytes with an extracellular flux analyzer showed some promising results. This led us to generate the TAC model and to administer citrulline in the model, but—contrary to our expectations—citrulline per se did not ameliorate cardiac dysfunction. To explore the possible underlying mechanisms, we conducted RNA sequencing analysis. Interestingly, in the EMPA group we found enrichment in the KEGG terms related to insulin signaling. Because of this enrichment, we considered it essential to characterize capillary network formation in heart after EMPA administration. In these studies, we found that capillary rarefaction developed, as previously reported^8,9^, and—interestingly—was enhanced in the TAC + EMPA group. We then re-analyzed the results from our metabolomic studies and found that the arginine level was significantly reduced in the TAC + EMPA group compared with the TAC group. This led us to consider that the finding of reduced arginine, together with an increased citrulline level, might suggest an enhanced metabolism from arginine to citrulline plus NO, which would be indicative of eNOS activation. Consistent with this notion, the NO level was significantly increased in cardiac tissues of the TAC + EMPA group compared with the TAC group. The studies with an NOS inhibitor, L-NAME, showed that co-administration of this compound enhanced capillary rarefaction and inhibited improvement of cardiac dysfunction in the TAC + EMPA group, indicating that the eNOS/NO axis is crucial for the cardioprotective effects of EMPA. Our results also suggest that NE overload increases ROS and reduces the pAkt signal, thereby inducing endothelial apoptosis, and that butyrate plays a critical role in inhibiting NE-induced ROS production and endothelial apoptosis. Our results suggested that EMPA activates the AKT/eNOS/NO pathway in ECs and that this activation contributes to the inhibition of apoptosis in ECs and maintenance of capillarization in cardiac tissues during LV pressure overload. Previous studies^17,30–32^ showed potential impacts of EMPA on endothelial function, and our study supplements these studies by providing novel insights into the beneficial effects of EMPA on the failing heart.

A recent study by Lopez et al. demonstrated that nNOS in cardiomyocytes increased with EMPA^33^, and cardiomyocyte-specific overexpression of nNOS was previously reported to improve cardiac function during LV pressure overload^34^. Our results and these previous reports indicate the existence of possible NO transduction between non-ECs and ECs, which may mediate their cardioprotective effects. Additional studies are needed to test the potential interaction between the nNOS/NO pathway in cardiomyocytes and the eNOS/NO pathway in endothelial cells.

Although it is well known that SGLT2 inhibitors induce ketogenesis in patients with type 2 diabetes, ketogenesis is not dominant in nondiabetic conditions and may not play an important role in nondiabetic heart failure^35^. Santos-Gallego et al.^28^ analyzed a nondiabetic pig myocardial infarction model and showed that EMPA ameliorated adverse left ventricular remodeling in nondiabetic heart failure by enhancing myocardial energetics through switching toward utilization of ketone body, free fatty acid, and branched-chain amino acid. In our study, EMPA treatment significantly altered circulating hydroxybutyrate levels in the TAC group but not in the Sham group (Fig. 2B). We previously demonstrated that heart failure could promote systemic insulin resistance and glucose intolerance by inducing visceral adipose tissue inflammation^15^, which may influence the effect of EMPA on plasma hydroxybutyrate levels in the TAC + EMPA group. Thus, we believe that SGLT2 inhibitors could exert cardioprotective effects by enhancing ketone utilization in nondiabetic patients with heart failure.

In conclusion, as demonstrated by previous other studies, EMPA activates various cardioprotective pathways, and our findings indicate a critical role of the AKT/eNOS/NO pathway in ECs for the maintenance of capillarization and suppression of pathologies in the failing heart.

## Methods

### Mice

All animal experiments were conducted in compliance with the guidelines which was reviewed by the Institutional Animal Care and Use Committee of Niigata University and this study is approved by the President of Niigata University. The study was carried out in compliance with the ARRIVE guidelines. We used wild-type male mice with a C57BL/6NCrSlc background. Transverse aortic constriction (TAC) was performed in these mice, as previously described^8^, at 9 weeks of age. After TAC, mice were maintained on a normal chow diet (CE-2, Clea, Japan) with or without EMPA (0.03% w/w). With this dose of EMPA, urine volume was significantly increased in the EMPA group, but we found no differences in body weight, renal function, or blood pressure between the Control and EMPA groups. Some groups of mice were fed CE-2 with EMPA for a total of 2 weeks after TAC, and other groups were fed CE-2 with 1.0% citrulline and given a daily intraperitoneal injection of citrulline for a total of 2 weeks (25 mg), starting 2 weeks after TAC. The animals were euthanized 2 weeks after TAC by intraperitoneal barbiturate injection, and tissues were quickly collected for further analyses. In some studies, L-NAME (1 mg/ml; Sigma-Aldrich, N5751) was administered in drinking water 2 weeks after TAC for a total of 2 weeks.

### Physiological analyses

Mice were housed at room temperature (23 to 24 °C). Echocardiography was performed with a Vevo 2100 High Resolution Imaging System 4 weeks after TAC (Visual Sonics Inc.), as previously reported^9,15^. Blood pressure was analyzed with a tail-cuff system, as previously described^36^. Urine was collected by housing mice individually in urine-collection cages for 24 h (KN-645, Natsume), and plasma and urine samples were analyzed by SRL Inc. Cardiac catheterization was performed by Primetech Life Science Laboratory, as previously reported^37^, with the following slight modifications: The sampling rate was 2 kHz, the version of iWorx analytic software was Labscribe4, and a 1.2F catheter from Tsansonic Inc. was used. For the perfusion study of cardiac tissues, a microsphere (Invitrogen F8803, FluoSpheres Carboxylate-Modified Microspheres, 0.1 µm, yellow-green fluorescent [505/515], 2% solids) was used. At euthanasia, cardiac tissues were perfused with normal saline with heparin (10 U/ml; total volume, 10 ml) and then with normal saline (5 ml) with 250 μl of the microsphere. Cardiac tissues were collected and homogenized with a tissue lyser in radioimmunoprecipitation assay (RIPA) lysis buffer (10 mM Tris-hydrochloride [HCl], pH 8, 140 mM sodium chloride [NaCl], 10 mM EDTA, 0.025% sodium azide [NaN3], 1% Triton X-100, 1% deoxycholate, 0.1% sodium dodecyl sulphate [SDS], and 4% protease inhibitor). After centrifuge, supernatant was collected and 200 μl of the solution was analyzed with a Nivo Multimode Microplate Reader (PerkinElmer), according to the manufacturer's instructions, at an excitation and emission of 480 nm and 530 nm, respectively. The relative signal was calculated and adjusted for the protein concentration in the solution.

### Metabolomic analyses

Metabolomic analyses were performed by T.S. and colleagues by capillary electrophoresis/mass spectrometry (CE/TOFMS), as previously described^38^.

### Histological analyses

Cardiac tissue samples were harvested, fixed overnight in 10% formalin, embedded in paraffin and sectioned for immunofluorescence, picrosirius red staining, or hematoxylin–eosin (HE) staining. In some studies, tissues were fixed in OCT compounds (Miles Inc.) and snap-frozen in liquid nitrogen to prepare cryostat sections. Fibrosis was detected with Picrosirius red stain, and 4 fields per section were randomly selected for quantification with the ImageJ system. TUNEL (terminal deoxynucleotidal transferase–mediated biotin–deoxyuridine triphosphate nick-end labeling) was performed according to the manufacturer’s protocol (MEBSTAIN Apoptosis TUNEL Kit Direct, MBL life science, code No.8445) and combined with isolectin GS-IB4 from Griffonia simplicifolia biotin-XX conjugate (Thermo Fisher Scientific, I21414) and Hoechst (Thermo Fisher Scientific, 33,258). In some studies, phospho eNOS (Thermo Fisher Scientific, PA5-105,824) and phospho-AKT1 (Ser473) recombinant rabbit monoclonal antibody (98H9L8; Thermo Fisher Scientific, 700,392) were co-stained with IB4 isolectin and cell membrane was detected with wheat germ agglutinin (WGA) Alexa Fluor 488 conjugate (Thermo Fisher Scientific #W11261). The number of IB4 isolectin-positive vessels was counted, and vascular density was estimated as the number of microvessels relative to the number of cardiomyocytes/cross-sectional area (CSA) (μm^2^), as previously reported^9^. NO production was evaluated by staining with diaminorhodamine-4 M acetoxymethyl ester (DAR-4 M, Goryo Chemical SK1006-01), as previously reported^39^. The secondary antibody for phosphor-eNOS antibody and phospho-AKT1 (Ser473) was goat anti-rabbit IgG H&L (Cy5; abcam, ab97077). For the study with CD31, anti-CD31 antibody (abcam [ab28364], 1:25) was used, followed by a Biotin-SP-AffiniPure Donkey Anti-Rabbit IgG (H + L) antibody (Jackson ImmunoResearch, 711-065-152, 1:50) and Cy5-streptavidin (Invitrogen, 43-4316, 1:50). All primary and secondary antibodies were used at a dilution of 1:50 (unless otherwise mentioned), except for Hoechst, which was used at a dilution of 1:1000. In some studies, reactive oxygen species (ROS) was evaluated with dihydroethidium (DHE) staining (WAKO, 041-28251). Stained sections were photographed with a Biorevo (Keyence Co.) or a confocal microscope (Nicon). For electron microscopy, heart tissue was fixed in 2.5% glutaraldehyde. Grids for electron microscopy were prepared by Mr. Masaaki Nameta at the core electron microscope facility of Niigata University, and electron microscopy was done at the Niigata University Medical Campus with a JEM1400 TEM, as previously reported^40^.

### Cell culture, chemicals and drugs

Human umbilical vein endothelial cells (HUVECs, Lonza) were cultured with Endothelial Cell Growth Medium-2 BulletKit (Lonza), and C2C12 cells (ATCC, CRL1772) were cultured with Dulbecco’s Modified Eagle Medium (DMEM) and differentiated according to the manufacturer’s instruction. Arginine (5 mM for a total of 73 h; Merck A5006), SC79 (AKT activator; 10 μM for a total of 73 h; abcam, ab146428), 3-hydroxybutyrate (3-HB; 10 mM for a total of 73 h; Sigma-Aldritch, 54,920), or DETA NONOate (a NO donor; 100 μM for 73 h; abcam, ab144627) was administered 1 h before norepinephrine (NE) was introduced (10 ng/ml for a total of 72 h; Daiichi Sankyo), and the medium containing these reagents was refreshed every 24 h. For the Western blot study for phosphor AKT detection, HUVECs were serum starved with 0.5% FBS for a total of 4 h; at the start of the starvation, 3-HB (10 mM Sigma-Aldritch, 54920) was added in some plates. One hour after the starvation, NE (10 ng/ml) was added for a total of 3 h. Insulin (HumulinR, Lilly, 100 nmol/l) was administered 10 min before collecting cells.

### Extracellular flux assay

The cellular oxygen consumption rate and extracellular acidification rate were measured with a Seahorse XF extracellular flux analyzer, according to the manufacturer’s instructions (Agilent). For further studies, citrulline (5 mM for 6 h, Merck, C7629) was administered to differentiated C2C12 cells.

### Western blot analysis

Western blot analysis was conducted as described previously^9^. Whole-cell lysates were prepared in RIPA lysis buffer (10 mM Tris-hydrochloride [HCl], pH 8, 140 mM sodium chloride [NaCl], 10 mM EDTA, 0.025% sodium azide [NaN3], 1% Triton X-100, 1% deoxycholate, 0.1% sodium dodecyl sulphate [SDS], and 4% protease inhibitor). The lysates (30 μg) were resolved by the SDS–polyacrylamide gel electrophoresis (PAGE) method, and proteins were transferred to a polyvinylidene fluoride (PVDF) membrane (Millipore). The PVDF membrane was incubated with the primary antibody and then with horseradish peroxidase-conjugated anti-rabbit IgG (Jackson Laboratories). The primary antibodies used for Western blotting (all at 1:1000) were phospho-Akt (Ser473) antibody (Cell Signaling #9271), Akt antibody (Cell Signaling #9272), and β-actin antibody (Cell Signaling, #4967); the secondary antibody (at 1:5000) was Peroxidase AffiniPure Goat Anti-Rabbit IgG (H + L) (Jackson ImmunoResearch, 111-035-003).

### RNA analysis

RNA analysis was conducted as described previously^15^. Total RNA (1 μg) was isolated from cell samples with RNA-Bee (TEL-TEST Inc.). Quantitative PCR was performed by using a Light Cycler 480 (Roche) with the Universal Probe Library and the Light Cycler 480 Probes Master (Roche), according to the manufacturer’s instructions, for all molecules except for the angiogenic form of *Vegfa*, *Vegfa*_*164a*_, which was detected with the SYBR Green real-time PCR Master Mixes (Roche), according to the manufacturer’s instructions. The mouse primers and their sequences were as follows:

*Adgre1*; 5’- GGAGGACTTCTCCAAGCCTATT-3’, 5’- AGGCCTCTCAGACTTCTGCTT-3’.

*Atp5f1a*; 5’-TCCATGCCTCTAACACTCGAC-3’, 5’-GACGTGTCAGCTCCCAGAA-3’,

*Ccl2*; 5’-CATCCACGTGTTGGCTCA-3’, 5’-GATCATCTTGCTGGTGAATGAGT-3’,

*Dnm1*; 5’-GCTAGTCCACGTTTCACCAGA-3’, 5’-TCCATGTGGCAGGGTCAT-3’,

*Fis1*; 5’-AGCTGGTGTCTGTGGAGGAT-3’, 5’-ATTGCGTGCTCTTGGACAC-3’,

*Itgax*; 5’-GAGCCAGAACTTCCCAACTG-3’, 5’-TCAGGAACACGATGTCTTGG-3’,

*Mfn1*; 5’-GTGAGCTTCACCAGTGCAAA-3’, 5’-CACAGTCGAGCAAAAGTAGTGG-3’,

*Mfn2*; 5’-CGAGGCTCTGGATTCACTTC-3’, 5’-CAACCAGCCAGCTTTATTCC-3’,

*Mtnd5*; 5’-AGCATTCGGAAGCATCTTTG-3’, 5’-TTGTGAGGACTGGAATGCTG-3’,

*Ndufa1*; 5’-TGATGGAACGCGATAGACG-3’, 5’-GCCAGGAAAATGCTTCCTTA-3’,

*Opa1*; 5’-ACCAGGAGAAGTAGACTGTGTCAA-3’, 5’-TCTTCAAATAAACGCAGAGGTG-3’, *Ppargc1a*; 5’-TGAAAGGGCCAAACAGAGAG-3’, 5’-GTAAATCACACGGCGCTCTT-3’,

*Ptprc*; 5’-GAGGTGTCTGATGGTGCAAG-3’, 5’-TGTATTCCACTAAAGCCTGATGAA-3’,

*Rplp0*; 5’-GATGCCCAGGGAAGACAG -3’, 5’-ACAATGAAGCATTTTGGATAA-3’,

*Tnf*; 5’-TCTTCTCATTCCTGCTTGTGG-3’, 5’-GGTCTGGGCCATAGAACTGA-3’,

*Vegfa*_*164a*_; 5’-CAGAAAATCACTGTGAGCCTTGTT-3’, 5’-CTTGGCTTGTCACATCTGCAA-3’.

*Irs2*; 5’- TGACTATACCGAGATGGCCTTT -3’, 5’- GAGGTGCCACGATAGGTTGT -3’.

*Pik3r1*; 5’- GACGGCACTTTCCTTGTCC -3’, 5’- TGACTTCGCCGTCTACCAC -3’.

*Ppara*; 5’- CTGAGACCCTCGGGGAAC -3’, 5’- AAACGTCAGTTCACAGGGAAG -3’.

*Ppp1r3a*; 5’- TTGGTTTGTCAAAAGAAGAGGAA -3’, 5’- TCTGGTGCTAGCAATGGTTCT -3’.

*Prkaa2*; 5’- CGACTACATCTGCAAACATGG -3’, 5’- CAGTAATCCACGGCAGACAG -3’.

*Prkab2*; 5’- CGGGAAAGGAGCACAAGAT -3’, 5’- GCTGCCAGGGTACAAACTCT -3’.

*Sorbs1*; 5’- AGAACTCCAGGACCGATGC -3’, 5’- GCGAGTCTTCCCAGATGC -3’.

*Comp*; 5’- CTGCTCCGAGACAGCTACG -3’, 5’- GGGTGAGCATTGCACTCA -3’.

*Egf*; 5’- GGGATGTGGGGGACTTACTAC -3’, 5’- TGGCTCATCACAAGGGTTC -3’.

*Erbb4*; 5’- AATGCTGATGGTGGCAAGA -3’, 5’- CATCACTTTGATGTGTGAATTTCC -3’.

*Spp1*; 5’- CCCGGTGAAAGTGACTGATT -3’, 5’- TTCTTCAGAGGACACAGCATTC -3’.

*Tlr4*; 5’- GGACTCTGATCATGGCACTG -3’, 5’- CTGATCCATGCATTGGTAGGT -3.

In some studies, the magnetic-activated cell sorting (MACS) technique was used to correct CD45-negative and CD31-positive cells, as previously described^41^. For this study, cardiac tissues were minced into small pieces with dissecting scissors and then incubated with 1.2 units/ml of Dispase II (EIDIA, Japan), 2 mg/ml type IV collagenase (Worthington, NJ), and 2 mM CaCl2 in PBS for 45 min at 37℃. Every 15 min, the samples were pipetted to fracture cell clumps and achieve dissociation into single cells. The tissue digestion factors were neutralized by adding DMEM 10% FBS. To eliminate connective tissue and fibers, the samples were passed through a cotton gauze and a nylon cell strainer (70 μm and 40 μm, BD Falcon, NJ, USA). Then, the samples were centrifuged at 300* g* for 5 min and re-suspended in MACS buffer (PBS with 1% FBS and 2 mM EDTA). The magnetic-activated cell sorting (MACS) technique was used to remove CD45^+^ cells from the collected cells, after which CD31^+^ cells were selected. MACS was performed with anti-CD45 and anti-CD31 microbeads. The antibodies purified rat anti-mouse CD45 (BD Pharmingen, Catalog No. 550539) and purified NA/LE rat anti-mouse CD31 (BD Pharmingen, Catalog No. 553369) were used for MACS studies. RNA was extracted from these cells for further studies.

### Fluorescence-activated cell sorting (FACS) analysis

Fresh cardiac samples were harvested from mice and minced into small pieces with dissecting scissors, then incubated with 1.2 u/ml of Dispase (Gibco), 2 mg/ml of type II collagenase (Worthington, NJ), and 1 mM of CaCl2 in PBS for 45 min at 37 °C. The tissue digestion factors were neutralized by adding DMEM (Sigma) containing 20% FBS. To eliminate connective tissue and fibers, the samples were passed through 70-μm nylon cell strainers (Corning). The samples were centrifuged at 500* g* for 5 min, re-suspended in FACS buffer (PBS with 1% FBS and 5 mM EDTA) and incubated with CD31-PE/Cy7 (1/100; Biolegend Catalog No. 102418) and CD45-PE (1/100; Biolegend Catalog No. 103106) antibodies in 100 μl of FACS buffer for 15 min at room temperature. After washing with FACS buffer, samples were used for the experiments described below.

To measure intracellular ROS level, we used CellRox DeepRed (Invitrogen, C10422) according to the manufacturer’s instructions. To count apoptotic cells, we incubated samples with Annexin V—FITC (25:1; BD Bioscience Catalog No. 556419) in 100 μl of Annexin V Binding Buffer (Becton Dickinson and Company [BD]) for 15 min at room temperature and then added 5 times the volume of FACS buffer to stop Annexin V conjugation. To measure phospho-Akt protein expression, we fixed samples with Lyse/Fix buffer (BD) at room temperature for 10 min and then permeabilized them with Perm Buffer II (BD) according to the manufacturer's instructions. Samples were then incubated with Phospho-Akt-Alexa Fluor 488 antibody (1/100; R&D Catalog No. IC7794G) in FACS buffer at room temperature for 30 min, washed and resuspended with FACS buffer. Before samples were loaded into a cell analyzer they were passed through 40-μm nylon cell strainers (Corning). Flow cytometry was performed with a spectral cell analyzer ID7000 (SONY) according to the manufacturer's instructions. Acquired data were analyzed with Flow Jo version 10.7.1 (BD).

### RNA sequencing analysis

Total RNA was isolated from tissue samples with an RNeasy Mini Kit (Qiagen, 74104) and its quality was assessed by using the Agilent 2100 Bioanalyzer with the Agilent RNA6000 pico Kit (Agilent Technologies). The TruSeq Stranded mRNA LT Sample Prep Kit (Illumina) was used to construct 4 libraries, according to the specifications of the manufacturer. Then, these libraries were analyzed on a NextSeq500 with a NextSeq 500/550 High Output Kit v2 (Illumina). TopHat 2 (version 2.0.13) was used for mapping reads to the reference genome (Ensembl GRCm38/mm10) with annotation data downloaded from the Ensembl Asia website (URL https://asia.ensembl.org/). The expression of each transcript was quantified as the number of fragments per kilobase of transcript per million fragments mapped (FPKM), and expression was compared between 3 groups by Cuffdiff (included in Cufflinks version 2.2.1). The gene expression data obtained were deposited in the Gene Expression Omnibus database (GSE157792). Heatmap was generated with R studio (version 1.1, powered by R version 3.5.2). Visualization of functional association networks among differentially expressed genes and GO analysis was performed with STRING (version 10.5, URL https://string-db.org/).

### Enzyme-linked immunosorbent assay

Citrulline was analyzed with a Mouse Citrulline Enzyme-Linked Immunosorbent Assay (ELISA) Kit (MyBioSource, MBS027373) according to the manufacturer's instructions.

### Statistical analysis

Statistical analyses were performed with SPSS version 24 (IBM Corp.). All samples studied were biologically different, and all values were analyzed unless stated otherwise. In some studies, outliers and abnormal values were excluded by boxplot analyses for further analyses; details are described in each figure legend. Analyses were performed with and without these values; when the results of both analyses were nonsignificant, the difference was described as NS. Data are shown as the mean ± SEM. Differences between groups were examined by a 2-tailed Student’s *t* test or two-way analysis of variance (ANOVA), followed by Tukey’s multiple comparison test, the non-parametric Kruskal Wallis test, or Dunnett’s test for comparisons among more than 2 groups. In all analyses, P < 0.05 was considered statistically significant.

## Supplementary Information

Supplementary Information.
