# Supplementary material for: Empagliflozin maintains capillarization and improves cardiac function in a murine model of left ventricular pressure overload
Source: Sci Rep. 2021 Sep 15;11:18384. doi: 10.1038/s41598-021-97787-2 (PMC8443662; doi:10.1038/s41598-021-97787-2)
Supplement: Supplementary file 1 — Supplementary Information. [file 41598_2021_97787_MOESM1_ESM.pdf]

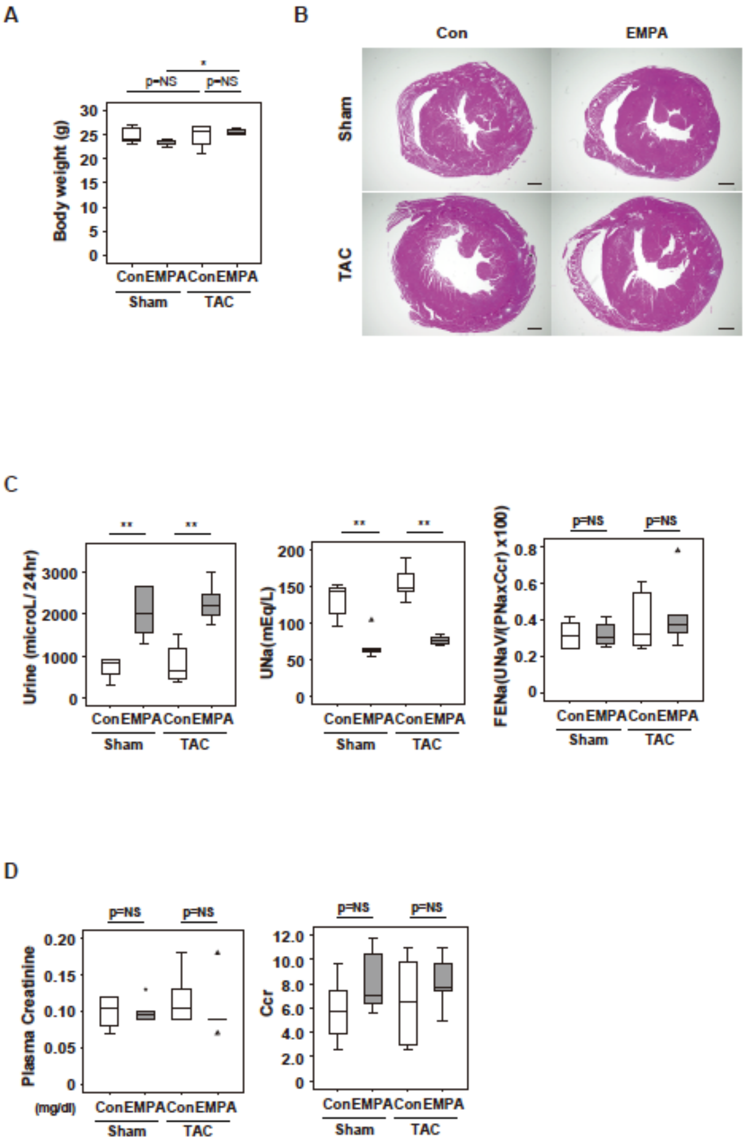

Supplemental Figure 1. Characterization of mice administered empagliflozin (EMPA)

(A, B): Body weight (A; n = 5, 5, 4, 4); HE staining of cardiac tissues (B; scale bar = 500 µm) of indicated group. (C, D) Urine volume (Urine)(n=6,6,6,6), urine sodium concentration (UNa)(n=6,6,6,6), fractional excretion of sodium (FENa)(n=6,6,6,6)(C), and plasma creatinine(n=6,6,6,6), creatinine clearance (Ccr)(n=6,6,6,6)(D) of indicated mice. Data were analyzed by a 2-way analysis of variance (ANOVA) followed by Tukey's multiple comparison test (A, C(UNa, FENa), D) or by non-parametric Kruskal Wallis test (C(Urine)). *$P < 0.05$, **$P < 0.01$. Values are shown as the mean ± SEM. NS = not significant. Small circle indicates outlier, triangle indicates abnormal value. Analyses were performed with and without these values; when the results of both analyses nonsignificant, the difference was described as NS.

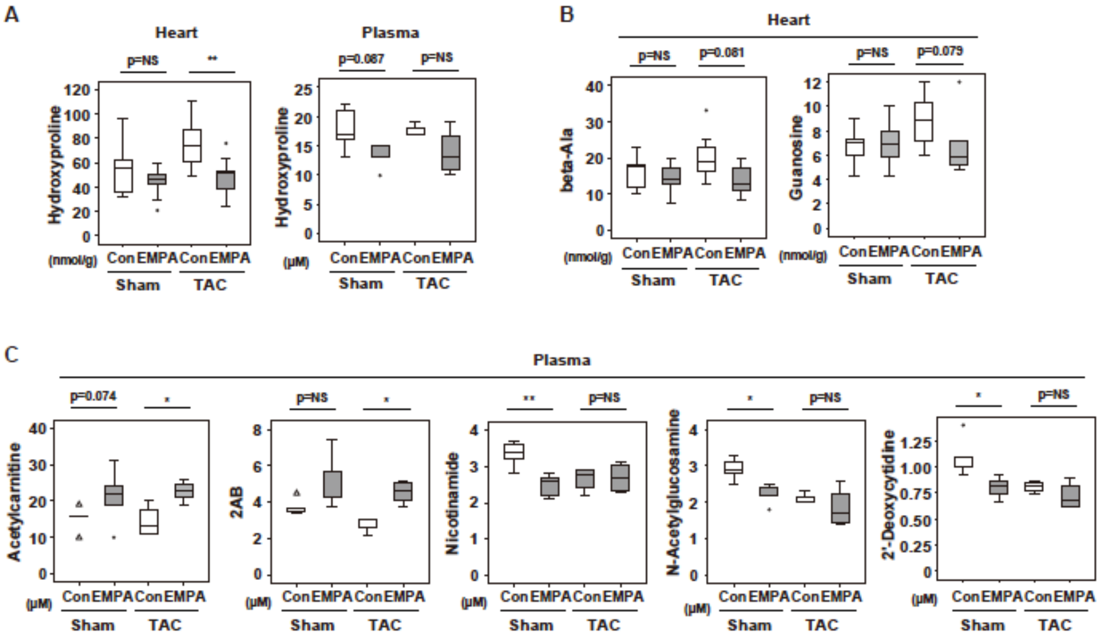

Supplemental Figure 2. Characterization of metabolites in heart and plasma with empagliflozin (EMPA) administration

(A, B): Level of hydroxyproline (A; left panel; n = 10, 10, 8, 9); and beta-alanine (beta-Ala) and guanosine (B; n = 10, 10, 8, 9) in heart of indicated groups. (A, C): Level of hydroxyproline (A; right panel; n = 5, 5, 4, 4); and acetylcarnitine, 2-aminobutyrate (2AB), nicotinamide, N-acetylglucosamine, and 2'-deoxycytidine (C; n = 5, 5, 4, 4) in plasma of indicated mice. Data were analyzed by a 2-way analysis of variance (ANOVA) followed by Tukey's multiple comparison test (A, B, C). The following were excluded for further analyses: in Sup. Fig. 2A, 1 outlier in TAC+EMPA group (heart) and 1 outlier in Sham+EMPA group (plasma); in Sup. Fig. 2B, 1 outlier in TAC+Con group; in Sup. Fig. 2C, for acetylcarnitine data, 2 abnormal values in Sham+Con group, 1 outlier in Sham+EMPA group; and for 2AB data, 1 abnormal value in Sham+Con group; in Sup. Fig. 2C, 2AB was analyzed by non-parametric Kruskal Wallis test. **P* < 0.05, ***P* < 0.01. Values are shown as the mean ± SEM. NS = not significant. Small circle indicates outlier, triangle indicates abnormal value. Analyses were performed with and without these values; when the results of both analyses were nonsignificant, the difference was described as NS.

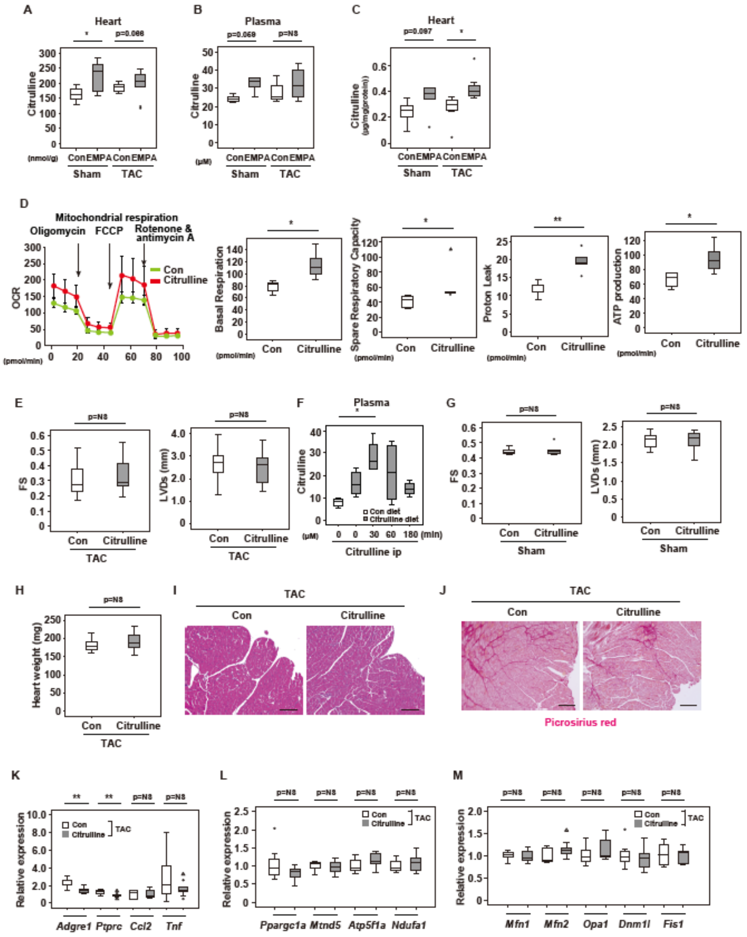

Supplemental Figure 3. Citrulline does not mediate beneficial biological effect of EMPA during LV pressure overload

(A, B): Metabolomic study analyzing citrulline in cardiac tissues (A; n = 10, 10, 8, 9) or plasma (B; n = 5, 5, 4, 4) of indicated mice. For study in Sup. Fig. 3A, 2 outliers (in the TAC+EMPA group) were excluded by boxplot (SPSS) for further statistical analysis (n = 10, 10, 8, 7 were analyzed). (C) ELISA study for citrulline in heart of indicated mice (n=5,5,7,7). (D) Evaluation of mitochondrial respiration with the Seahorse extracellular flux analyzer in differentiated C2C12 cells incubated with phosphate-buffered saline or citrulline (5mM, 6 hours; n = 5, 5). For analysis of spare respiratory capacity, 1 abnormal value in the citrulline group was excluded for further analysis (n = 5, 4 were analyzed). (E) Echocardiographic data analyzing fractional shortening (FS) or left ventricular systolic dimension (LVDs) of mice subjected to TAC or Sham operation with or without (Con) citrulline administration (n = 25, 27). (F) ELISA study for citrulline in plasma of mice administrated with control diet or citrulline diet. In some citrulline diet groups, mice were also injected with citrulline intraperitoneally (ip). The time 30, 60, 180 min indicates the time when blood samples were collected after ip administration of citrulline (n=4,4,4,4,4). (G)Echocardiographic data analyzing fractional shortening (FS) or left ventricular systolic dimension (LVDs) of indicated mice (n = 7, 7). (H) Heart weight of indicated mice (n = 7, 9). (I, J): Hematoxylin and eosin (HE) staining (I) or picrosirius red staining (J) of left ventricle (LV) of indicated mice. Scale bar = 100 µm. (K, L, M): Quantitative PCR of *Adgre1* (n = 7, 9), *Ptprc* (CD45; n = 7, 9), *Ccl2* (n = 7, 9), and *Tnf* (K; n = 6, 9); *Ppargc1a, Mtnd5, Atp5f1a,* and *Ndufa* (L; n = 7, 9); and *Mfn1, Mfn2, Opa1, Dnm1l,* and *Fis1* (M; n = 7, 9) of indicated mice. Data were analyzed by a 2-tailed Student's *t* test (D, E, G, H, K-M) or 2-way ANOVA followed by Tukey's multiple comparison test (A, C, F) or Dunnett's test (B). **P* < 0.05, ***P* < 0.01. Values are shown as the mean ± SEM. NS = not significant. Small circle indicates outlier, triangle indicates abnormal value. Analyses were performed with and without these values; when the results of both analyses were nonsignificant, the difference was described as NS.

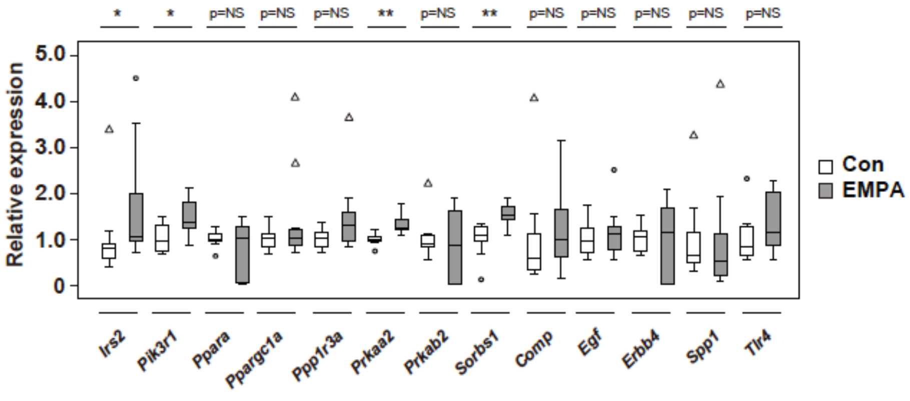

Supplemental Figure 4. Quantitative PCR analyses of insulin-signaling related molecules

Quantitative PCR studies analyzing *Irs2*, *Pik3r1*, *Ppara*, *Ppargc1a*, *Ppp1r3a*, *Prkaa2*, *Prkab2*, *Sorbs1*, *Comp*, *Egf*, *Erbb4*, *Spp1* and *Tlr4* in the cardiac tissues of indicated groups (n=10, 11). For this study, cardiac tissues were collected 5 days after the introduction of EMPA at TAC2w, and samples were collected at TAC2w+5days.

Data were analyzed by a 2-tailed Student's *t* test. $*P < 0.05$, $**P < 0.01$. Values are shown as the mean ± SEM. NS = not significant. Small circle indicates outlier, triangle indicates abnormal value. Analyses were performed with and without these values; when the results of both analyses were nonsignificant, the difference was described as NS.

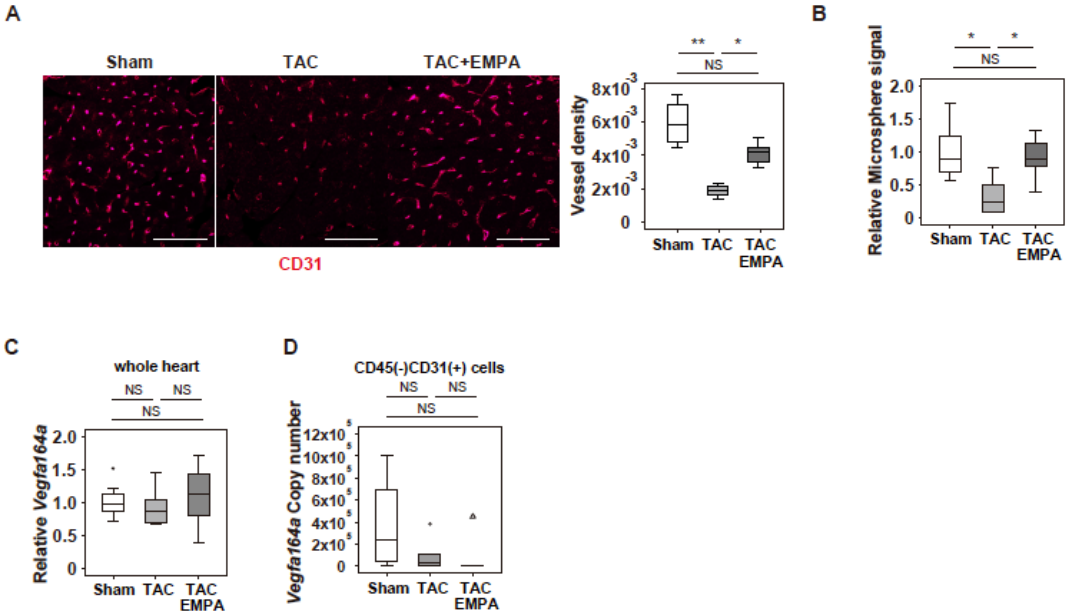

Supplemental Figure 5. A role of angiogenesis and endothelial apoptosis in empagliflozin (EMPA)-induced cardiac protection

(A) Immunofluorescence study for CD31 in cardiac tissues of indicated mice. Right panels indicates quantified data demonstrated as vessel density (estimated as the number of microvessels relative to the number of cardiomyocytes/ cross sectional area (CSA) (μm^2))(n=4,5,5(A), scale bar = 100μm). (B) Relative microsphere signal from the cardiac tissues of the indicated mice (n=6,6,6). (C) Quantitative polymerase chain reaction (PCR) of *Vegfa164a* in whole heart of indicated group (n = 8, 9, 9). (D) Copy number of *Vegfa164a* in CD45$^-$CD31$^+$ cells in heart of indicated group (n = 4, 5, 5). Data were analyzed by 2-way analysis of variance (ANOVA) followed by non-parametric Kruskal Wallis test (A), Tukey's multiple comparison test (B, C), Dunnett correction (D). **P* < 0.05, ***P* < 0.01. Values are shown as the mean ± SEM. NS = not significant. Small circle indicates outlier, triangle indicates abnormal value. Analyses were performed with and without these values; when the results of both analyses were nonsignificant, the difference was described as NS.

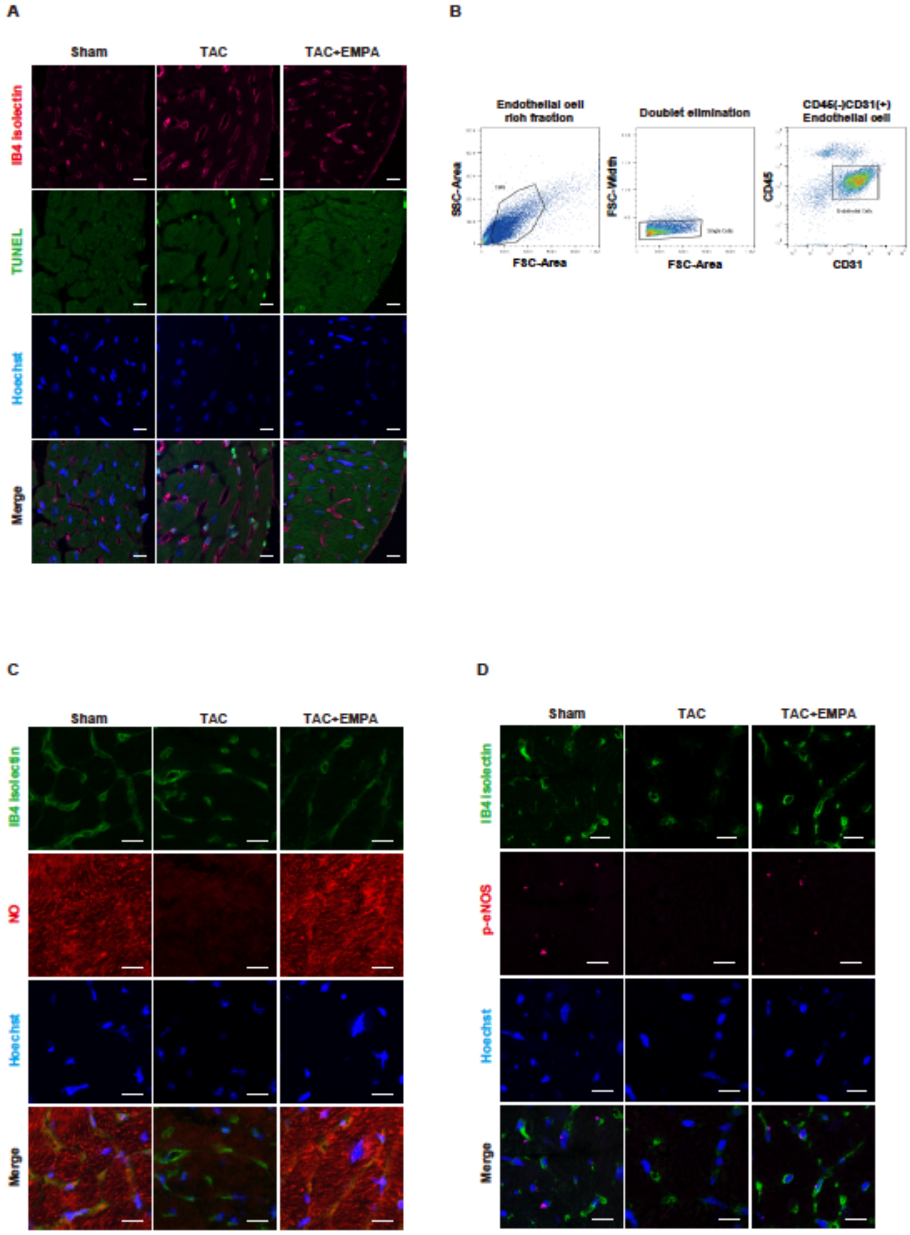

Supplemental Figure 6. Empagliflozin (EMPA) enhances capillarization in left ventricle during pressure overload

(A, C, D): IB4-isolectin staining co-stained with TUNEL (terminal deoxynucleotidal transferase–mediated biotin–deoxyuridine triphosphate nick-end labeling) and Hoechst (A; scale bar = 10 µm); NO and Hoechst (C; scale bar = 10 µm); p-eNOS and Hoechst (D; scale bar = 10 µm). (B) Flow-cytometry panels showing the method of gating $CD45^{-}CD31^{+}$ endothelial cells[ECs].

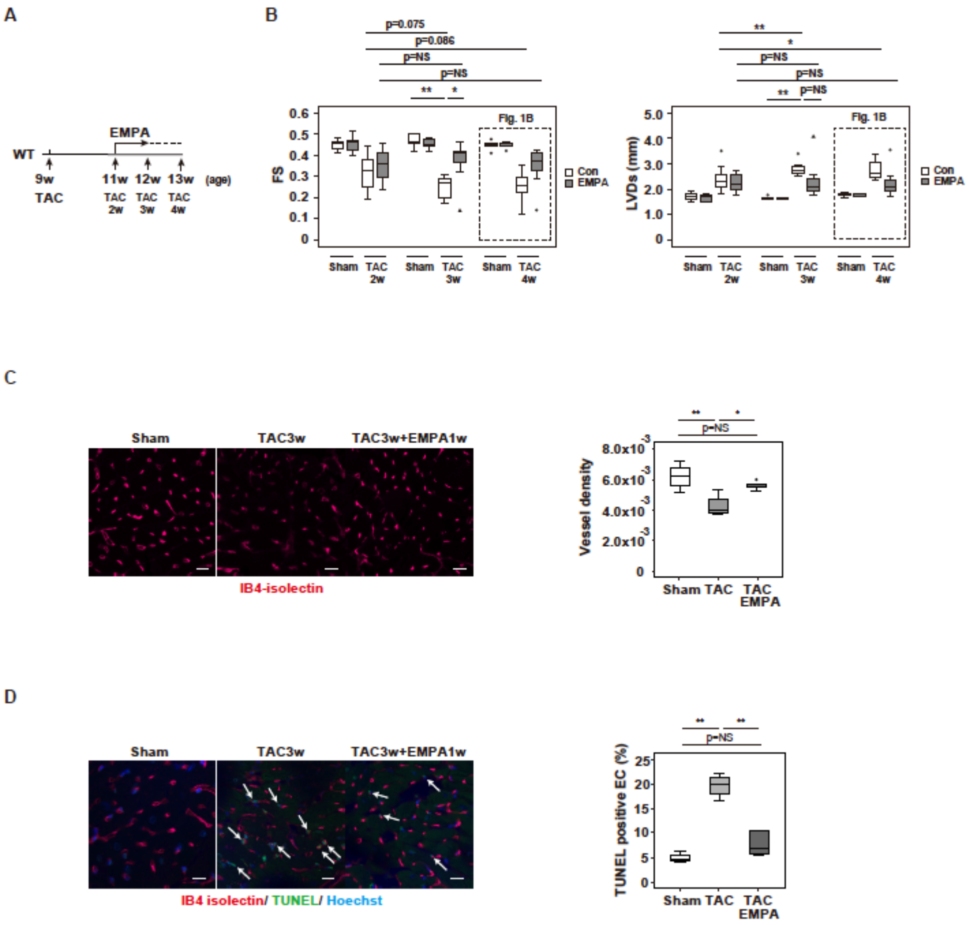

Supplemental Figure 7. Temporal studies with empagliflozin administration

(A)Protocol of empagliflozin(EMPA) administration. At 9 weeks of age, mice were subjected to TAC operation. Two weeks after TAC, some mice were subjected to EMPA administration for totally 1 weeks and euthanized at 12 weeks of age (3 weeks after TAC operation). (B) Echocardiographic data analyzing fractional shortening (FS) or left ventricular systolic dimension (LVDs) of mice subjected to TAC or Sham operation with or without (Con) administration of empagliflozin (EMPA) (n = 5, 5, 14, 7(for TAC2w studies), n = 5, 5, 8, 7(for TAC3w studies), n = 5, 5, 8, 7(for TAC4w studies)(data for TAC4w are also presented in Fig. 1B). For this study, 1 outlier (in the TAC2w group (Con)) was excluded by boxplot (SPSS) for further statistical analysis. (C) Capillarization of left ventricle (LV) of indicated mice as analyzed with IB4-isolectin staining. Right panel indicates vessel density (n = 4, 4, 5). Scale bar = 10 µm. (D) IB4-isolectin staining co-stained with TUNEL and Hoechst (scale bar = 10 µm; arrow indicates TUNEL-positive endothelial cells [ECs]) Right panel indicates TUNEL positive EC (%) (n = 4, 4, 5). Data were analyzed by 2-way analysis of variance (ANOVA) followed by Tukey's multiple comparison test. *$P < 0.05$, **$P < 0.01$. Values are shown as the mean ± SEM. NS = not significant. Small circle indicates outlier, triangle indicates abnormal value. Analyses were performed with and without these values; when the results of both analyses were nonsignificant, the difference was described as NS.
